# Mouse feeding study and microbiome analysis of sourdough bread for evaluation of its health effects

**DOI:** 10.3389/fmicb.2022.989421

**Published:** 2022-09-21

**Authors:** Joon-Gi Kwon, Sung-Hoon Park, Jeong-Eun Kwak, Jae Hyoung Cho, Gooyoun Kim, Deukbuhm Lee, Dong Hyun Kim, Hyeun Bum Kim, Ju-Hoon Lee

**Affiliations:** ^1^Department of Agricultural Biotechnology, Seoul National University, Seoul, South Korea; ^2^Department of Food and Animal Biotechnology, Seoul National University, Seoul, South Korea; ^3^Center for Food and Bioconvergence, Seoul National University, Seoul, South Korea; ^4^Research Institute of Agriculture and Life Sciences, Seoul National University, Seoul, South Korea; ^5^Department of Food and Nutrition, Gangneung-Wonju National University, Gangneung, South Korea; ^6^Department of Animal Resource Science, Dankook University, Cheonan, South Korea; ^7^Research Institute of Food and Biotechnology, SPC Group Co., Seoul, South Korea

**Keywords:** sourdough, fermentation, microbiome, mouse feeding study, health promotion

## Abstract

Sourdough bread fermented with yeast and lactic acid bacteria (LAB) is thought to have various beneficial health effects. However, its beneficial effects were not fully evaluated with *in vivo* mouse model. To evaluate these effects *in vivo*, a mouse feeding study and microbiome analysis of white bread containing 40% sourdough (WBS) and yeast-leavened white bread (WB) were performed. Although feed consumption and body weight increased with WBS, the glycemic index was reduced, suggesting a diabetes-lowering effect, probably due to the presence of dietary fiber and short-chain fatty acids (SCFA). In addition, a mineral absorption test showed that WBS increased magnesium absorption owing to phytate degradation during fermentation. Interestingly, WBS decreased total cholesterol and triglycerides, probably due to the dietary fiber and SCFA in LAB. In addition, the ratio of low- and high-density lipoprotein was decreased in WBS, implying potential risk reduction for cardiovascular disease. An immunomodulatory assay of WBS revealed that pro-inflammatory cytokines TNF-α and IL-6 were decreased, suggesting anti-inflammatory activity. Gluten degradation by fermentation and antioxidation activity of menaquinol/ubiquinol by gut microbiota also supported the anti-inflammatory activity of sourdough bread. Furthermore, some beneficial gut bacteria, including *Akkermansia*, *Bifidobacterium*, and *Lactobacillus*, were increased in WBS. In particular, *Akkermansia* has been associated with anti-inflammatory properties. Consequently, WBS has beneficial effects on health, including decreased glycemic index and cholesterol, increased mineral availability and absorption, anti-inflammatory properties, and establishment of healthy gut microbiota.

## Introduction

Fermented foods have been consumed for thousands of years, and functional studies have been conducted to evaluate their beneficial health effects in humans. Food fermentation enhances flavor and taste as well as health-promoting functionalities, in addition to extending food preservation for food safety. In particular, it decomposes undesirable food components and nutrient absorption inhibitors in the ingredients of raw food materials (Marco et al., 2021). Yeast-fermented bread made of wheat flour is one of the most important staple foods consumed worldwide (Dewettinck et al., 2008). Although this yeast-fermented white bread is a primary source for energy production, excessive consumption may be associated with diabetes or celiac disease (CD) owing to its high glycemic index (GI), high gluten levels, and low fiber content resulting from the refinement of wheat flour containing high levels of carbohydrate (Koistinen et al., 2018; Demirkesen-Bicak et al., 2021). To reduce these, whole grain cereal-based bread that has not undergone a refining process is sometimes consumed. However, it is not highly palatable to consumers due to its undesirable texture, dark color, and bitter taste after baking (Jiang and Peterson, 2013; Heiniö et al., 2016). Recently, sourdough-based breads have attracted public interest because of their potential health-promoting effects. Sourdough is generally made of wheat flour and then fermented with yeast as well as lactic acid bacteria (LAB) (Arora et al., 2021). LAB fermentation endows better flavor and taste and also enhances digestion via decomposition of undesired ingredients in wheat flour. Recent studies have revealed that the consumption of sourdough bread alleviates some nutritional problems potentially caused by white bread consumption, such as elevated postprandial blood sugar levels and triglyceride levels (Gil-Cardoso et al., 2021; Pagliai et al., 2021). However, the functional mechanism of these effects of consumption of sourdough bread is not yet completely understood. Therefore, the aim of this study was to understand and evaluate these health-promoting functionalities of sourdough bread using an *in vivo* mouse model to examine nutrition, decreases in GI and cholesterol, digestion efficiency, mineral absorption, immune response, and gut microbiota regulation.

Recently, with the development of metabolomics, sourdough bread was found to contain more organic acids, including lactic acid, total amino acid, and mannitol, than yeast-fermented bread (Shewry et al., 2022). These results are helpful for the study of the gut microbiome after consuming sourdough bread. In addition, changes in pH, hydrolysis of proteins and indigestible carbohydrates, and production of metabolites such as short-chain fatty acids (SCFAs) during sourdough fermentation improve the functionality of sourdough. For example, during sourdough fermentation, pH reduction by LAB enhances phytase activity; degrades phytate; and chelates minerals such as iron, calcium, and magnesium; thereby increasing the bioavailability of calcium, magnesium, iron, and zinc (Lopez et al., 2001; Frontela et al., 2011; Gibson et al., 2018). Moreover, CD is one of the most common food intolerances. When gluten, a protein present in grains such as wheat, rye, and barley, is consumed, patients with CD recognize it as an antigen, resulting in autoimmune disease. Therefore, gluten-free products are essential for CD patients. However, gluten is found in all wheat species, including common wheat, durum, spelt, Khorasan, emmer, and einkorn, and is therefore, present in many bread products. Although gluten is known as the causative agent of CD, an autoimmune enteropathy, it is hydrolyzed during sourdough fermentation (Thiele et al., 2004; Gujral et al., 2012). Therefore, these studies demonstrated potential health benefits of sourdough.

The study of microbiota changes during sourdough fermentation was performed based on next-generation sequencing technology. Many sourdough microbiome studies have been conducted in the past, such as monitoring of changes in the microbiota and metabolic profiling of sourdough during the fermentation process by region and wheat variety (Alfonzo et al., 2017; Koistinen et al., 2018), along with changes in the gut microbiota after ingestion of microorganism isolated from sourdough (Liu et al., 2020; Zhong et al., 2021). Fewer comparative studies on changes in the gut microbiota of the host when consuming yeast-fermented bread and sourdough bread have been conducted because of the assumption that they do not influence the gut microbiota since the microorganisms are killed by high temperatures during the baking process. However, organic acids such as lactic acid, SCFAs, and metabolites produced during sourdough fermentation are known to have beneficial effects on health (Wong et al., 2006; Canfora et al., 2015). Therefore, the purpose of our study was to explore how sourdough bread changes the gut microbiota and how these changes alter their physiological properties. Thus, glucose, mineral, and cholesterol levels in blood or serum and cytokine levels in colon tissue were determined, and microbiome analysis was performed to determine its correlation with physiological features.

## Materials and methods

### Bread making procedure and sample preparation

For the bread preparation process, whole baking ingredients were provided by SPC Co. (South Korea). The breadmaking process was slightly modified from traditional methods: wheat flour (Mildawon, Inc., South Korea), whole wheat flour (Mildawon, Inc.), malt, salt, yeast, and tap water were mixed and stored overnight at 4°C. After fermentation at low temperature, the first fermentation was carried out at 12°C for 90 min, the fermented dough was molded, and the second fermentation was performed at 27°C for 100 min. The bread was then baked for 25 min. Sourdough was prepared with wheat flour and whole wheat flour and then fermented at 27°C overnight with a yeast (*Saccharomyces cerevisiae* SPC-SNU 70-1; SPC) and SPC Health Guard™ (a mixture of four lyophilized LAB; *Lactobacillus brevis* SPC-SNU 70-2, *L. curvatus* SPC-SNU 70-3, *Fructilactobacillus sanfranciscensis* SPC-SNU 70-4, and *L. plantarum* SPC-SNU 72-1). WB was prepared without sourdough, and sourdough bread was prepared with 40% sourdough, following the breadmaking procedure. White bread (without sourdough), sourdough bread (40% sourdough added), and sourdough (not baked) were lyophilized and homogenized for mouse feeding.

### Animals, diets, and study design

**FIGURE 1:**
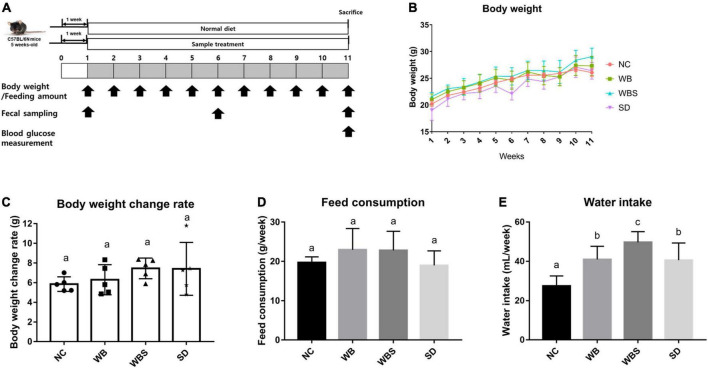
**(A)** Overall experimental schedule of mouse feeding study with a one-week adaptation period and 10-week feeding period. Body weight and feeding amount were measured every week. Feeding samples were collected at weeks 1, 6, and 11. Blood glucose was measured at week 11. **(B)** Body weight measurement of the *in vivo* mouse models each week. NC: normal diet group as a negative control, WB: mouse feeding group with yeast-fermented white bread, WBS: mouse feeding group with 40% sourdough-supplemented white bread, SD: mouse feeding group with 100% unbaked sourdough. **(C)** Body weight change ratio for weeks 1–11 in each group. **(D)** Average of feed consumption amount every week in each group. **(E)** Average of water intake every week in each group. Error bars present the standard deviations of five replicates (*n* = 5 male mice) in each group. One-way ANOVA followed by Duncan’s *post-hoc* analysis, and bars with different letters indicate significant differences at *p* < 0.05.

All experiments using mice were approved by the Institutional Animal Care and Use Committee of Dankook University (Cheonan, Korea; approval no. DKU-20-018) and conducted in accordance with Care and Use of Laboratory Animals guidelines. Male C57BL/6 mice (5 weeks old) were purchased from Raon Bio Co. (South Korea) and randomly divided into four groups (*n* = 5/group): control group (NC; normal chow diet with AIN-76A, Envigo, USA), white bread group (WB; yeast-leavened bread diet), white bread containing 40% sourdough group (WBS; 40% sourdough-containing bread diet), and sourdough group (SD; 100% sourdough, not baked), before starting the feeding study. During the first week, all mice were acclimatized to laboratory conditions consisting of a 12:12 h light/dark cycle at 24°C and 55% humidity with a normal chow diet. The feeding study was performed for 10 weeks after the one-week adaptation period. During the feeding period, all defined samples were fed *ad libitum* to all four groups of mice, and the remaining samples, water intake, and body weight were determined each week. Fecal samples were collected at weeks 1, 6, and 11. The mice were euthanized and blood and colon samples were collected at week 11 for an *ex vivo* test (Figure 1A). The blood samples were obtained from the submandibular vein of the mice using a lancet and centrifuged at 2,000 × *g* for 10 min, and then the supernatant was separated to obtain the serum. All fecal, blood, and colon samples were stored at –80°C until further testing.

### Glycemic index *in vivo* test

Three days before euthanasia, the mice were fasted for 12 h for the GI *in vivo* test. After a 12 h fasting period, a chow diet and bread sample for all four groups were fed to the mice, and then the tail tip of the mice was cut off. Five blood samples in each group were collected at 0, 30, 60, 90, and 120 min. All collected blood samples were used to determine the glucose levels in the samples with an Accu-Chek Performa blood glucose meter (Roche Diabetes Care, Switzerland). The GI was determined using the area under the curve (AUC) calculation method (Chlup et al., 2008).

### Determination of iron, calcium, and magnesium in mouse serum

The serum of mice was used to determine the concentrations of iron, calcium, and magnesium using an iron assay kit (Cat. #ab83366; Abcam, UK), calcium assay kit (Cat. #ab102505l; Abcam), and magnesium assay kit (Cat. #ab102506; Abcam), respectively. All experiments were performed following the manufacturer’s standard protocol.

### Cholesterol levels in mouse serum

The mouse serum was also used to determine the total cholesterol levels as the sum of high-density lipoprotein (HDL), low-density lipoprotein (LDL), and triglycerides (TG). The measurements of HDL/LDL and TG were performed using a cholesterol assay kit (Cat. #ab65390; Abcam) and a triglyceride assay kit (Cat. #ab65336; Abcam), respectively. All experiments were conducted according to the manufacturer’s standard protocol.

### Cytokine assay

To determine cytokine levels in the colon, colonic tissue lysates were prepared by cutting the colon of each mouse to 1 cm and then homogenizing with a bead beater (Bead Ruptor Elite, OMNI International, USA) in RIPA buffer (150 mM sodium chloride, 50 mM Tris-HCl, pH 7.4, 1 mM ethylenediaminetetraacetic acid, 1 mM phenylmethylsulfonyl fluoride, 1% Triton X-100, 1% sodium deoxycholic acid, 0.1% sodium dodecyl sulfate, and 5 μg/mL of leupeptin; ATTO, Japan). After centrifugation of colonic tissue lysate at 12,000 × g for 1 min, the supernatant was collected and used to estimate cytokine levels of tumor necrosis factor (TNF)-α (Cat. #K0331186P; Koma Biotech, South Korea), interleukin (IL)-6 (Cat. #K0331230; Koma Biotech), and IL-10 using an ELISA kit (Cat. #K0331213P; Koma Biotech), respectively.

### Microbiome analysis

#### Total DNA extraction from mouse fecal samples

Fecal samples from mice in each group were collected at weeks 1, 6, and 11. Total DNA of the fecal samples was extracted using a QIAamp Fast DNA Stool Mini Kit (Cat. #51604; Qiagen, USA), according to the manufacturer’s instructions. Extracted fecal DNA was quantified by a NanoDrop 2000 (Thermo Scientific, USA) and adjusted to a concentration of 5 ng/μL using UltraPure water (Cat. #ML 019-02; WELGENE, South Korea).

#### Preparation for MiSeq analysis

Diluted total fecal DNA (5 ng/μL) was used for PCR amplification with a universal primer set targeting from the V3 to V4 region of the 16S rRNA gene (341F, 5′-CCT ACG GGN GGC WGC AG-3′; 805R, 5′-GAC TAC HVG GGT ATC TAA TCC-3′). The amplification conditions using KAPA HiFi HotStart Ready Mix (Cat. #07958935001; Roche, Switzerland) were as follows: 1 cycle at 95°C for 3 min, 35 cycles at 95°C for 30 s, 35 cycles at 55°C for 30 s, 35 cycles at 72°C for 1 min, and 1 cycle at 72°C for 5 min. Then, the PCR amplicons were extracted using an AxyPrep DNA Gel Extraction kit (Cat. #AP-GX-250; Corning, USA). The amplicon library preparation was performed using a Nextera XT DNA Library Preparation Kit (Cat. #FC-131-1096; Illumina, USA) and the amplicons were sequenced using the Illumina MiSeq 2 × 300 bp paired-end sequencing method (Sanigen, Inc., South Korea).

#### Bioinformatic analysis

The raw FASTQ files were imported and processed using QIIME 2 (v. 2020.8) (Bolyen et al., 2019), and forward and reverse reads were joined using the q2-vsearch plugin, sequentially denoising with Deblur to remove and correct noisy reads. For taxonomic analysis, representative sequences were classified based on the SILVA database (version 132) (Quast et al., 2013) using the q2-feature-classifier plugin. To understand the impact of each sample on the gut microbiota, β-diversity analysis was performed using redundancy analysis (RDA) based on the Bray-Curtis dissimilarity of each sample. To compare the difference in abundances of individual taxonomic groups among sample groups, differential abundance analysis was performed using the DESeq2 package in R (Love et al., 2014). Additionally, the microbial functional feature was performed using PICRUSt 2.0 (Phylogenetic investigation of communities by reconstruction of unobserved states) in the q2-picrust2 plugin (Douglas et al., 2020). Additional analyses and visualization were performed using R v3.6.1 (Ihaka and Gentleman, 1996) with the microbiome R package (Lahti and Shetty, 2017) and STAMP (Parks et al., 2014).

### Statistical analysis

Data were analyzed using GraphPad Prism version 7.0.0 software (GraphPad, USA), SPSS version 26.0 (IBM, USA) and R version 3.6.1 and presented as means ± standard deviation. The statistical analysis was performed by ANOVA test for comparison of multiple groups and by Student’s *t*-test for comparison of two groups. Differences were defined as significant at *p* < 0.05.

## Results

### Body weight, feed consumption, and water intake

The body weight change, feed consumption, and water intake of all groups were determined each week after the adaptation period. The body weights increased for whole feeding period, but all samples increased at a similar ratio (Figure 1B). To clarify the body weight changes in all groups, body weight change rates were calculated and compared by subtracting the weight from week 11 to week 1 in each sample, which revealed that the WBS group had the greatest weight change (Figure 1C). To clarify the weight change of all groups, the weight change rate was calculated and compared by subtracting the weight from the 11th week to the 1st week from each sample. Although their comparative analysis was not statistically significant, the average of body weight change in WBS group was the highest among them. However, the WB and WBS groups showed similar feed consumption, suggesting that the digestion efficiency of sourdough bread might be better than that of white bread, resulting in higher weight change in the WBS group (Figure 1D). In addition, water intake amounts of the WB, WBS, and SD groups were much higher than that of the NC group, probably due to lyophilization of the feeding samples (Figure 1E).

### The glucose concentration in the blood and glycemic index

**FIGURE 2:**
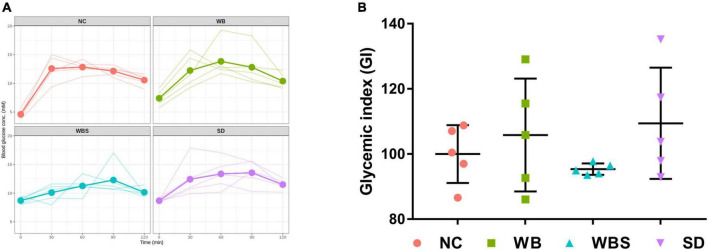
Change in blood glucose concentration and glycemic index (GI) in all groups. **(A)** Line graph of blood glucose concentration of individual mouse in all groups up to 2 h. Bold line presents the average value of five replicates (*n* = 5 male mice) in each group. **(B)** GI dot plot with error bar of five replicates (*n* = 5 male mice) in each group. NC: normal diet group as negative control, WB: mouse feeding group with yeast-fermented white bread, WBS: mouse feeding group with 40% sourdough-supplemented white bread, SD: mouse feeding group with 100% unbaked sourdough.

To evaluate the effects of consumption of sourdough bread on blood glucose levels, the concentrations of blood glucose in all groups were determined. The changes are illustrated in Figure 2A. While the fasting blood glucose concentration of NC group was lower than that of the other group, the change rate of blood glucose concentrations was lowest in the WBS group, indicating a low impact on blood glucose level by sourdough bread (Figure 2A). To verify this, GI was determined for each group using an AUC calculation (Figure 2B). Interestingly, the GI of the WBS group was also much lower than that of other groups, such as the change rate of blood glucose concentration in WBS group. A previous report showed that sourdough bread is a low GI food that increases blood glucose level slowly after ingestion (Capurso and Capurso, 2020), which supports this finding.

### Mineral concentration in mouse blood

**FIGURE 3:**
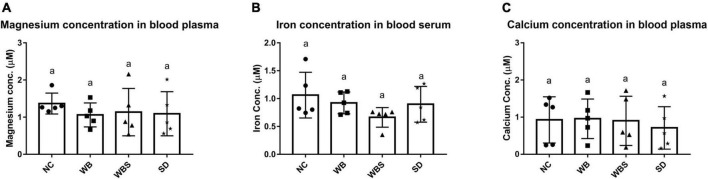
Mineral bioavailability and absorption assay. **(A)** Magnesium concentration in mouse blood plasma in each group. **(B)** Iron concentration in mouse blood serum in each group. **(C)** Calcium concentration in mouse blood plasma in each group. Error bars present the standard deviations of five replicates (*n* = 5 male mice) in each group. One-way ANOVA followed by Duncan’s *post-hoc* analysis, and bars with different letters indicate significant differences at *p* < 0.05.

To evaluate the influence of sourdough bread ingestion for mineral absorption, the blood concentrations of minerals for all groups were determined and compared. There were no statistically significant differences in the concentrations of any minerals in the mouse blood (Figure 3). However, the concentrations of magnesium and iron in blood samples for WB, WBS, and SD groups were slightly lower than that of NC group (Figures 3A,B). In particular, the iron concentration of WBS is the lowest among them (Figure 3B). Although consumption of sourdough bread and body weight increased in WBS group, it is not clearly understood why adsorption and blood concentration of iron decreased. To further understand this, it is necessary to elucidate interaction or competition between the mouse host and its microbiota for iron uptake and utilization, because gut bacteria also require iron for survival and energy metabolism in the gut environment. Therefore, the question regarding iron uptake and utilization was further studied in the functional microbiome analysis of mouse gut microbiota in the WBS group. Interestingly, the magnesium concentration of the WBS group was slightly higher than that of the WB and SD groups, suggesting that absorption and utilization of magnesium in WBS group may be enhanced, probably due to fermentation with yeast and LAB. However, there was no significant difference in the concentration of calcium among the NC, WB, and WBS groups, even though the calcium concentration of the SD group was lower than that of the other groups (Figure 3C).

### Cholesterol levels in mouse serum

**FIGURE 4:**
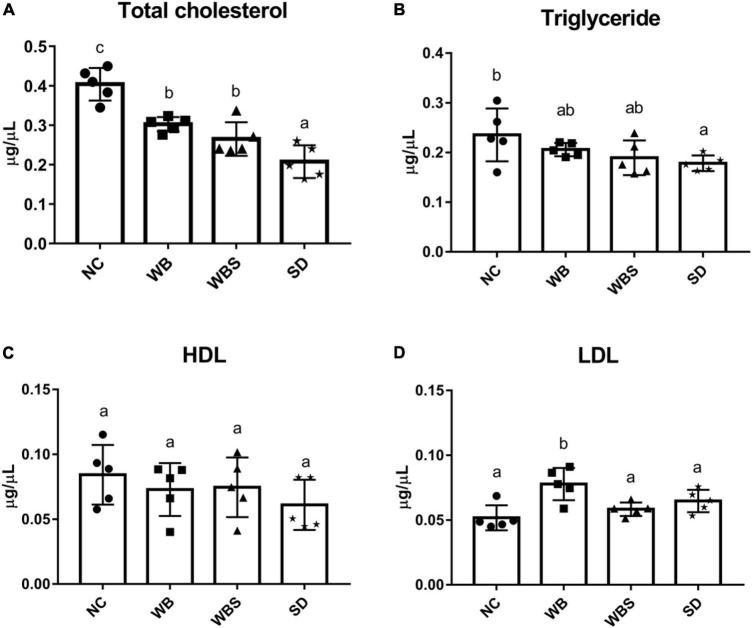
Cholesterol assay in mouse blood serum of all groups. **(A)** Total cholesterol assay. **(B)** Triglyceride assay. **(C)** High-density lipoprotein (HDL) assay. **(D)** Low-density lipoprotein (LDL) assay. Error bars present the standard deviations of five replicates (*n* = 5 male mice). One-way ANOVA followed by Duncan’s *post-hoc* analysis, and bars with different letters indicate significant differences at *p* < 0.05.

A previous report found that consumption of fermented sourdough bread could reduce the cholesterol level in the serum (Capurso and Capurso, 2020). To verify this, total cholesterol, HDL, LDL, and TG were quantified in serum samples of all groups. When NC, WB, WBS, and SD samples were consumed, amounts of total cholesterol, TG, and HDL in serum samples of the WB, WBS, and SD groups were reduced compared to that of the NC group, and were lowest in the SD group (Figures 4A–C). However, LDL in serum samples of the WB, WBS, and SD groups was higher than that in the NC group (Figure 4D). Interestingly, the ratio of LDL and HDL in the WBS group was much lower than that in the WB and SD groups, probably involved in the reduction of cardiovascular disease (Figures 4C,D).

### Immune response

**FIGURE 5:**
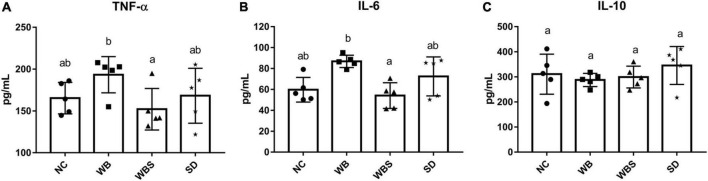
Cytokine assay of colon tissue of the *in vivo* mouse model in each group. **(A)** TNF-α. **(B)** IL-6. **(C)** IL-10. Error bars present the standard deviations of five replicates (*n* = 5 male mice). One-way ANOVA followed by Duncan’s *post-hoc* analysis, and bars with different letters indicate significant differences at *p* < 0.05.

To evaluate this immunomodulatory effect of sourdough bread, the production of inflammatory cytokines in the gut of each group was determined. The levels of pro-inflammatory cytokines TNF-α and IL-6 were significantly increased in the WB group but decreased significantly in the WBS group compared to the NC and WB groups (Figures 5A,B). However, there was no statistically significant difference in the production of an anti-inflammatory cytokine IL-10 (Figure 5C). The different cytokine production patterns between the WB and WBS groups indicate the reduction of gluten in the WBS group by sourdough bread fermentation, suggesting possible alleviation of CD by consumption of fermented sourdough bread.

### Microbiome analysis

#### Diversity and composition analysis of the mouse gut microbiota

**FIGURE 6:**
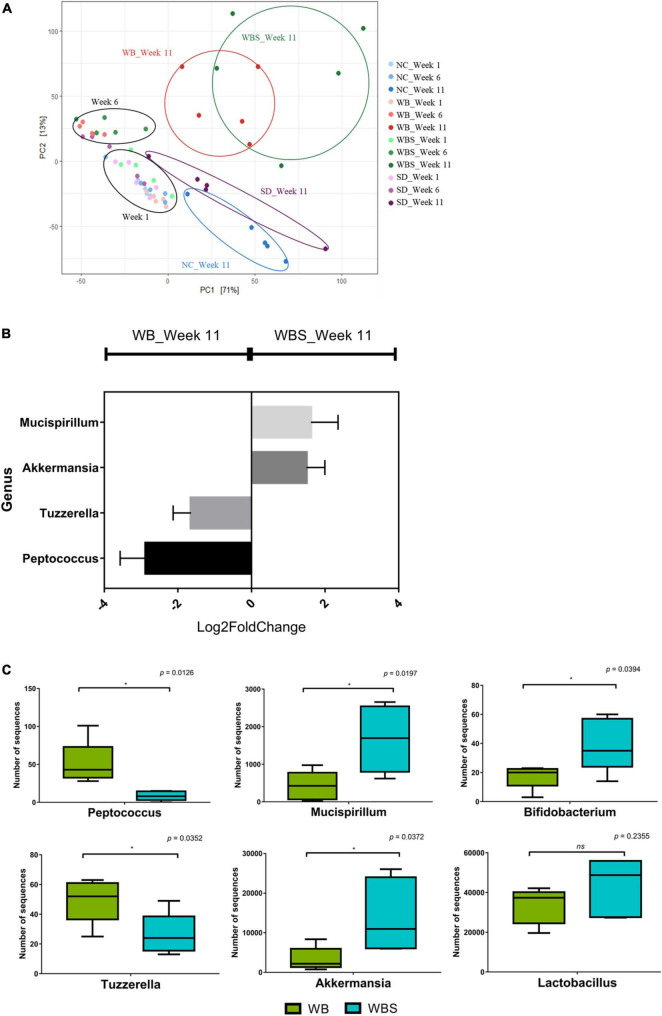
Microbiome analysis of fecal samples from the *in vivo* mouse model of all groups. **(A)** Redundancy analysis (RDA) plot of each group at weeks 1, 6, and 11 using Bray-Curtis dissimilarity. **(B)** Differential abundance analysis in genus level using DESeq2. **(C)** Boxplot of comparative composition analysis of *Peptococcus*, *Tuzzerella, Mucispirillum*, *Akkermansia, Bifidobacterium*, and *Lactobacillus* at week 11 in the WB and WBS groups. WB: mouse feeding group with yeast-fermented white bread, WBS: mouse feeding group with 40% sourdough-supplemented white bread.

The response and alteration of mouse gut microbiota by feeding of bread samples and their correlation with previous results are important to understanding the role of bread consumption in nutrition and health. To perform this microbiota study, mouse fecal samples were collected at weeks 1, 6, and 11 from each group. The average sequencing depth of all sequencing samples was 232,867.65 which was found to have sufficient sequencing depth as the line slopes for the observed features converge to zero (Supplementary Figure 1). The β-diversity analysis revealed that there was no significant difference between weeks 1 and 6 (Figure 6A). However, gut microbiota in all groups were altered and separated at week 11, suggesting that long-term intake may be necessary for change of gut microbiota. Notably, after alteration and separation of the groups during week 11, there were two different directions in the diversity graph: baked bread groups (WB and WBS) and unbaked groups (NC and SD) (Figure 6A). Therefore, the baking process may be important for functional differentiation of sourdough between WBS and SD.

To understand the role and function of sourdough in the gut microbiota, a comparative analysis of the WB group (white bread without sourdough; yeast-fermented) and WBS group (fermented sourdough bread; yeast and LAB-fermented) was conducted. Comparative composition analysis of gut microbiota in these groups showed no significant difference, except for *Akkermansia* in the WBS group at week 11 (Supplementary Figure 2). However, differential abundance analysis of mice gut microbiota between the WB and WBS groups at week 11 revealed that their group-specific genera were quite different. WB group-specific genera included *Peptococcus* and *Tuzzerella*, while WBS group-specific ones included *Mucispirillum*, and *Akkermansia* (Figures 6B,C). In addition, to confirm the sensitization effect of beneficial bacteria, *Bifidobacterium*, and *Lactobacillus* were identified. There were significant differences between the two groups in *Bifidobacterium*, but not in *Lactobacillus*.

#### Predictive functional profiling

**FIGURE 7:**
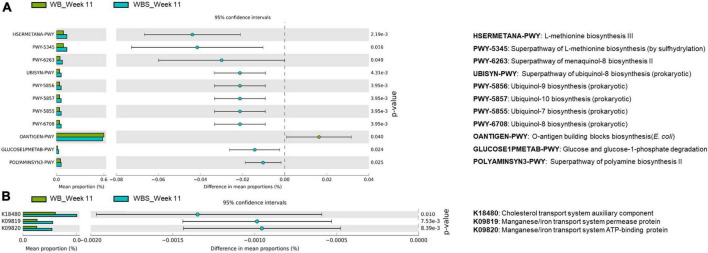
Predicted functional profiling analysis of WB and WBS groups at week 11 using PICRUSt2. **(A)** Prediction pathways and **(B)** functional orthologs using the KEGG database are illustrated on the extended error bar plot. WB: mouse feeding group with yeast-fermented white bread, WBS: mouse feeding group with 40% sourdough-supplemented white bread.

As explained, sourdough has an impact on the response and modulation of gut microbiota via LAB fermentation. To further understand the function of the bread samples with regard to modulated gut microbiota, comparative functional profile analysis of the gut microbiota between the WB and WBS groups at week 11 was performed. This analysis revealed two different key patterns: (1) the pentose phosphate pathway and O-antigen biosynthesis pathway were upregulated in the WB group, and (2) menaquinol/ubiquinol biosynthesis pathways and methionine/polyamine biosynthesis pathways were upregulated in the WBS group (Figure 7A).

Upregulation of the methionine and polyamine pathways in the WBS group is probably involved in gut homeostasis (Figure 7A). Based on these results, ingestion of LAB-fermented sourdough may be associated with possible anti-inflammation and gut homeostasis for gut health.

It is noteworthy that cholesterol transport and metal ion transport systems were upregulated in the WBS group, according to additional KEGG metabolic pathway orthology analysis. This analysis revealed that the cholesterol transport system was upregulated in the WBS group (Figure 7B). Previously, the amounts of total cholesterol and TG in the WBS group were lower than those in the WB group (Figures 4A,B), suggesting that this cholesterol transport system may be involved in the reduction of total cholesterol in the blood by ingestion of fermented sourdough bread. In addition, the bacterial manganese/iron transport system was upregulated in the WBS group, suggesting that iron uptake of gut microbiota in the WBS group was increased (Figure 7B). This dominant uptake and utilization of iron by gut bacteria implies the possible lowering of iron concentration in mouse blood due to competition between the mouse and gut bacteria for iron uptake and utilization (Figure 3B).

## Discussion

While white bread made of refined wheat flour is commonly consumed as a primary food for supplementation of nutrition and energy, problematic effects such as diabetes and CD have been suggested, probably due to its high carbohydrate, high GI, high gluten levels, and low dietary fiber (Koistinen et al., 2018; Demirkesen-Bicak et al., 2021). However, sourdough bread is generally supplemented with whole wheat flour and fermented with LAB. Therefore, it has low GI, low gluten levels, and high dietary fiber with better flavor and more organic acids due to LAB fermentation, resulting in relatively high consumer preference and health-promoting effects (Shewry et al., 2022). Although many studies have reported these beneficial effects of sourdough bread, there are relatively few functional studies on sourdough bread using *in vivo* evaluation and microbiome analysis. In this study, a comparative *in vivo* mouse feeding study and correlated microbiome analysis were performed to verify the health-promoting effects of sourdough bread compared to white bread.

The *in vivo* mouse feeding trials showed that sourdough bread increased feed consumption and body weight, but reduced GI (Figures 1C,D, 2B). Although total carbohydrates of white bread and sourdough bread are similar, total amino acids are increased in sourdough bread by gluten fermentation and degradation, suggesting that the amino acids produced in sourdough bread (not carbohydrates) may contribute to body weight gain but low GI (Rizzello et al., 2019). In addition, production of good flavor and organic acids by LAB fermentation may increase the preference for sourdough bread, which is probably associated with high consumption of sourdough bread and body weight gain. However, it is not clearly understood whether GI differs between sourdough bread and sourdough itself (not baked) (Figure 2B). A previous study reported that the bread baking process slightly increased total dietary fiber, probably contributing to lower GI (Caprez et al., 1986).

GI and total cholesterol have been shown to be positively associated, indicating that GI could be a risk factor for cardiovascular disease (Amano et al., 2004), which supports the patterns of GI, total cholesterol, TG, and HDL in the WB and WBS groups in this study. The amounts of total cholesterol and TG in WBS group were lower compared to the WB group, but the amount of HDL in the WBS group was slightly higher compared to the WB group (Figure 4). Therefore, the low GI of sourdough bread might be related to the regulation of cholesterol in mouse blood. In addition, cholesterol reduction is associated with SCFA and bacterial extrapolysaccharides (EPS) as dietary fiber (Fechner et al., 2014; Jurášková et al., 2022). Because sourdough bread is fermented with LAB, it produces additional SCFA and EPS during fermentation. Therefore, ingesting LAB-fermented sourdough bread may have a cholesterol-lowering effect due to LAB-produced SCFA and EPS. Furthermore, comparative functional profiling analysis revealed that the cholesterol transport system was upregulated in the WBS group (Figure 7B), which suggests that it may be involved in the reduction of total cholesterol by sourdough bread ingestion.

Wheat flour contains phytate and inhibits the absorption of minerals by chelation (Weaver and Kannan, 2002). Previous evidence that the enzyme activity of phytase from yeast increases during bread fermentation because this enzyme decomposes phytate in bread and reduces the mineral chelating activity of phytate supports this finding (Leenhardt et al., 2005). In this study, the determination of minerals (magnesium, iron, and calcium) in mouse blood plasma showed a reduction in mineral absorption compared to the NC group (Figure 3). Therefore, it is important to reduce phytate to increase mineral utilization efficiency for health. While yeast, a fermenter for bread making, has phytase to degrade phytate in wheat flour, this enzyme prefers acidic conditions (optimum pH is 5.5 or lower), so yeast phytase may be not be able to fully degrade phytate in white bread. However, fermentation with yeast and LAB yields more acidic conditions in sourdough bread, indicating that optimum phytate degradation activity in the WBS group might be higher compared to the WB group. Therefore, it was initially thought that mineral absorption efficiency in WBS group should be higher than that in the WB group. However, *in vivo* mineral absorption in the WB, WBS, and SD groups did not differ, except for iron absorption in WBS group. Phytates can be degraded during baking process and hydrolyzed during *in vivo* bread digestion in the stomach and small intestine (Lopez et al., 2003). Therefore, after ingestion of bread, phytates could be reduced during digestion, supporting no significant difference in *in vivo* mineral absorption between the WB and WBS groups in the present study (Figures 3A,C). Furthermore, comparative microbiome analysis between WB and WBS revealed upregulation of bacterial manganese/iron transport system in WBS group (Figure 7B). This result suggests that reduction of iron absorption to the mouse blood may be due to the competition between the mouse and the gut bacteria. In general, gut bacteria also require iron for survival and energy metabolism in the gut environment, supporting this.

A low-fat diet and dietary fiber can reduce total cholesterol and TG in the blood (Turley et al., 1998; Knopp et al., 1999). Therefore, reduction of total cholesterol and TG might be due to the low amount of total fat in the feeding samples of the WB, WBS, and SD groups compared to the NC group (Supplementary Table 1). In addition, the feed samples of WB, WBS, and SD groups were prepared with whole wheat flour containing dietary fibers, supporting this finding (see Materials and Methods). While the HDL levels in the WB, WBS, and SD groups were slightly lower, probably due to reduction of TG, the LDL levels of these groups with feeding of high carbohydrate bread samples were higher than that of the NC group (Figures 4B,D; Supplementary Table 1). High carbohydrate may be associated with increased LDL in the blood (Siri and Krauss, 2005), which supports this result. The ratio of LDL and HDL is generally considered a parameter for the risk of cardiovascular disease. Interestingly, the ratio of LDL and HDL in the WBS group was much lower than that in the WB and SD groups, suggesting that fermented sourdough bread may reduce the risk of cardiovascular disease (Figures 4C,D). Low GI is probably associated with the improvement of the LDL/HDL ratio (Jenkins et al., 1985). Based on this, the low GI of the WBS group might be correlated with reduction of the ratio (Figure 2B).

While white bread is high in gluten, the gluten level in sourdough bread is lower because it is digested by microorganisms during fermentation, which suggests that sourdough bread may be associated with anti-inflammatory effects by reducing the risk of CD. To evaluate the immune regulation function of sourdough bread, immunomodulatory cytokines were measured. Pro-inflammatory cytokines TNF-α and IL-6 were increased in the WB group but decreased in the WBS group, suggesting anti-inflammatory activity by sourdough bread (Figures 5A,B), even though there was no significant difference in anti-inflammatory cytokine IL-10 between the WB and WBS groups (Figure 5C). As previously mentioned, gluten is a CD-causing compound related to inflammation, and it is known that gluten is decomposed by sourdough fermentation (Thiele et al., 2004; Di Cagno et al., 2008). Therefore, reduction of gluten in sourdough bread may be associated with this anti-inflammatory activity. Further functional profiling analysis with microbiome data revealed that menaquinol and ubiquinol biosynthesis were activated in the WBS group, which suggests that they may reduce inflammation by their antioxidation activities (Rauscher et al., 2001; Schoon et al., 2001; Kuwabara et al., 2009; Amin et al., 2014; Figure 7A). Menaquinol, known as vitamin K2, is found mainly in foods fermented by LAB. Menaquinone, an oxidative form of menaquinol, is rare in patients with inflammatory bowel disease or Crohn’s disease (Schoon et al., 2001; Kuwabara et al., 2009). In addition, ubiquinol, called coenzyme Q, is known to have antioxidant and antidiabetic functionality (Rauscher et al., 2001; Amin et al., 2014). Therefore, menaquinol and ubiquinol may be involved in antioxidation resulting in anti-inflammatory effects in the gut environment. Down-regulation of O-antigen biosynthesis in WBS group compared to the WB group also supports this anti-inflammatory function (Figure 7A). Reduction of pro-inflammatory cytokines (TNF-α and IL-6) in previous immune response analyses also support this finding (Figures 5A,B). Polyamine, which consists of methionine as one of the main components, may play a role in maintaining the integrity of the gut epithelium via regulation of gut epithelial renewal and barrier function (Rao et al., 2020).

In addition, activation of methionine and polyamine biosynthesis in the WBS group was probably associated with maintenance of the integrity of gut epithelium for homeostasis. Therefore, antioxidation activity and maintenance of the gut epithelium might be involved in this reduction of inflammation. Dominance of *Akkermansia* in the gut microbiota of the WBS group may also support this inflammatory reduction (Figure 6C), because it plays an important role in the recovery and maintenance of the mucus layer in gut epithelial cells (Everard et al., 2013).

Comparative gut microbiota analysis showed that the difference in group-specific bacterial composition between WB and WBS groups may be related to sourdough fermentation by LAB. Mono-polysaccharides and oligosaccharides such as dextran and fructan are produced from whole grain flour in sourdough by LAB fermentation (Tieking and Gänzle, 2005; Galle, 2013), so sourdough bread generally has a high amount of fructan (Shewry et al., 2022). This fructan, including inulin, could account for the level of *Akkermansia* in the mouse gut microbiota (Everard et al., 2013; Zoetendal and Smidt, 2018), supporting its dominance in the WBS group. In addition, while the function of WB group-specific bacteria is not well-characterized, WBS group-specific bacteria are known to be associated with gut health promotion and gut microbiota regulation by production of organic acids and SCFA. In addition, comparative composition analysis of the gut microbiota in the WB and WBS groups revealed that ingestion of sourdough bread increased levels of some beneficial bacteria, including *Akkermansia, Bifidobacterium*, and *Lactobacillus* in the WBS group (Figures 6B,C). These bacteria are suggested to have health-promoting effects regarding the recovery of healthy gut microbiota via regulation of gut pathogens, alleviation of diarrhea and constipation, immune regulation, anti-carcinogenesis, and cholesterol reduction (Lee and O’Sullivan, 2010; Cheng and Xie, 2021). In particular, these bacteria are also related to production of SCFA and EPS, so are likely related to the reduction of GI and cholesterol. Therefore, sourdough bread has a positive impact on the modulation of gut microbiota with regard to health effects.

This *in vivo* mouse study showed potential health benefits of sourdough bread such as cholesterol reduction, inflammation alleviation, and health gut microbiota maintenance. However, it is still necessary to evaluate these health-promoting functions of sourdough bread by further human feeding trials and related clinical and more omics analysis in the future.

## Data availability statement

The datasets presented in this study can be found in online repositories. The names of the repository/repositories and accession number(s) can be found below: NCBI Sequence Read Archive (SRA) under accession number: PRJNA825082.

## Ethics statement

This animal study was reviewed and approved by the Institutional Animal Care and Use Committee of Dankook University (Cheonan, South Korea; approval no. DKU-20-018).

## Author contributions

S-HP, HK, and J-HL: conceptualization. J-GK and S-HP: methodology and formal analysis. J-GK, S-HP, J-EK, and J-HL: validation. J-GK, J-EK, and JC: investigation. S-HP, GK, DL, and DK: resources. J-GK, S-HP, and J-HL: writing—original draft. J-GK, HK, and J-HL: writing—review and editing. HK and J-HL: supervision. J-HL: project administration and funding acquisition. All authors contributed to the article and approved the submitted version.
