# Supplementary material for: Mouse feeding study and microbiome analysis of sourdough bread for evaluation of its health effects
Source: Front Microbiol. 2022 Sep 21;13:989421. doi: 10.3389/fmicb.2022.989421 (PMC9532698; doi:10.3389/fmicb.2022.989421)
Supplement: Supplementary file 1 [file Data_Sheet_1.docx]

**Supplementary Materials**

Mouse Feeding Study and Microbiome Analysis of Sourdough Bread for Evaluation of Its Health Effects

**Joon-Gi Kwon^1,2,3,4,†^, Sung-Hoon Park^5, †^, Jeong-Eun Kwak^1,2,3,4^, Jae Hyoung Cho^6^, Gooyoun Kim^7^, Deukbuhm Lee^7^, Dong Hyun Kim^7^, Hyeun Bum Kim^6^, and Ju-Hoon Lee^1,2,3,4,*^**

*^1^ Department of Agricultural Biotechnology, Seoul National University, Seoul, South Korea*

*^2^ Department of Food and Animal Biotechnology, Seoul National University, Seoul, South Korea*

*^3^ Center for Food and Bioconvergence, Seoul National University, Seoul, South Korea*

*^4^ Research Institute of Agriculture and Life Science, Seoul National University, Seoul, South Korea*

*^5^ Department of Food and Nutrition, Gangneung-Wonju National University, Gangneung, South Korea*

*^6^ Department of Animal Resources Science, Dankook University, Cheonan, South Korea*

*^7^ Research Institute of Food and Biotechnology, SPC Group, Seoul, South Korea*

**^†^ These authors have contributed equally to this work and share first authorship**

*** Corresponding author:**

Dr. Ju-Hoon Lee / juhlee@snu.ac.kr

**Table S1.** Nutrition composition of NC^a^, WB^b^, and WBS^c^ samples for mouse feeding

|  | NC (%) | WB (%) | WBS (%) |
| --- | --- | --- | --- |
| Protein | 20.30 | 16.16 | 15.49 |
| Carbohydrate | 70.00 | 79.09 | 78.91 |
| Fat content | 5.20 | 1.83 | 2.76 |
| Ash content | 4.50 | 2.92 | 2.84 |
| Total | 100 | 100 | 100 |

^a^, NC: normal diet group as negative control.

^b^, WB: mouse feeding group with yeast-fermented white bread.

^c^, WBS: mouse feeding group with 40% sourdough-supplemented white bread

**
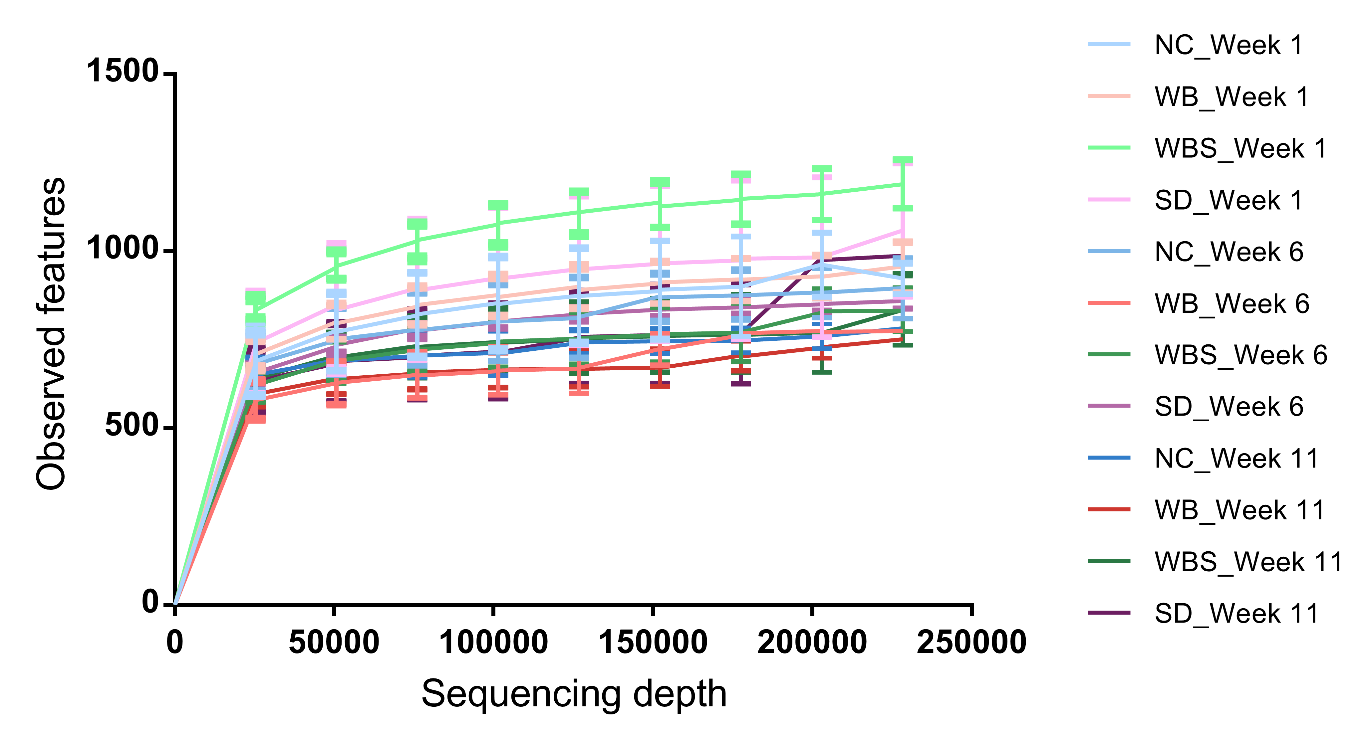
**

**FIGURE S1.** Alpha-rarefaction curve of microbiome analysis of fecal samples from *in vivo* mouse model of all groups.


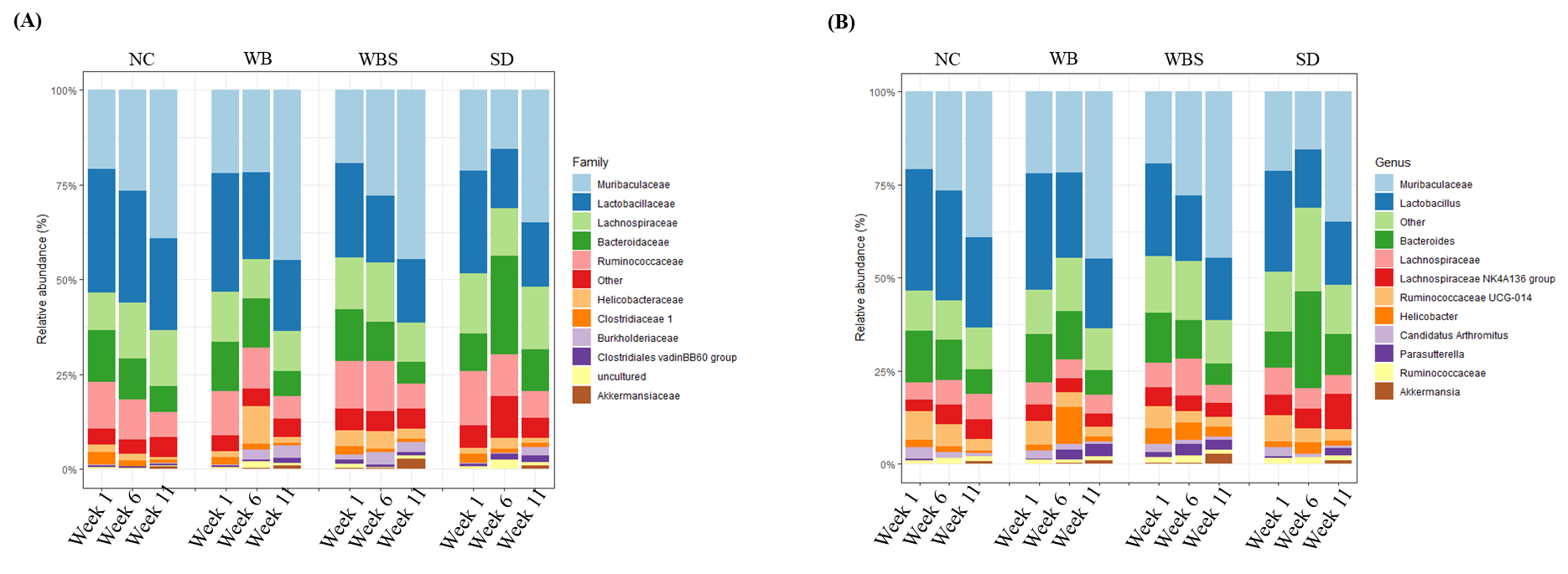


**FIGURE S2.** Composition and relative abundance of gut microbiota of *in vivo* mouse model in (A) family level and (B) genus level at week 1, 6, and 11
